# Constructing an Urban Population Model for Medical Insurance Scheme Using Microsimulation Techniques

**DOI:** 10.1155/2012/232071

**Published:** 2012-03-01

**Authors:** Linping Xiong, Lulu Zhang, Weidong Tang, Yuqin Ma

**Affiliations:** ^1^Department of Health Services Management, Second Military Medical University, Shanghai 200433, China; ^2^National Centre for Social and Economic Modelling, University of Canberra, Canberra, ACT 2601, Australia; ^3^Institute of Military Health Services Management, Second Military Medical University, Shanghai 200433, China; ^4^Bureau of Labour and Social Security of Kunming, Kunming 650011, China

## Abstract

China launched a pilot project of medical insurance reform in 79 cities in 2007 to cover urban nonworking residents. An urban population model was created in this paper for China's medical insurance scheme using microsimulation model techniques. The model made it clear for the policy makers the population distributions of different groups of people, the potential urban residents entering the medical insurance scheme. The income trends of units of individuals and families were also obtained. These factors are essential in making the challenging policy decisions when considering to balance the long-term financial sustainability of the medical insurance scheme.

## 1. Introduction

Medical care is one of the most important aspects in the social security system and plays an essential role in people's daily lives. In 1998, on the basis of several rounds of pilot programs and experimental implementation, the Chinese Government established a nationwide medical insurance system for the urban employed [1, 2]. For the vast rural areas, China founded a new cooperative medical care system in 2002.

In September 2007, China launched a pilot project of medical insurance to cover 240 million urban residents outside the workforce, and all urban residents would become beneficiaries by 2010 [3]. The pilot project has been carried out in 79 cities, including large cities as well as county-level cities [4]. As one of the pilot cities, Kunming (capital city of Yunnan Province) began its urban residents' medical insurance scheme in October 2007. For making their policy decisions, the government needs to have the information of the population distribution of different groups of people, employed individuals who are not covered by the medical insurance scheme, potential urban residents entering the medical insurance scheme in the coming years. The government also needs to know how much responsibility should the government take in the medical insurance scheme, especially for making subsidies for concessional individuals and families.

To answer these questions, an urban population model was created for China's medical insurance scheme using microsimulation model techniques. It focuses on the urban medical insurance system reform, involving population development for both urban employees and nonworking residents. The key aims of the research can be described in at least three aspects. First, it makes it clear for the policy makers the population distributions of different groups of people. Second, it estimates the potential urban residents entering the medical insurance scheme. Third, it creates a population model for helping to predict medical insurance policy distributional effects on urban individuals and families.

The main method used in this research is microsimulation. A microsimulation model differs from other types of models in that it operates on individual units rather than on aggregate information. The simulation model applies a set of rules to each individual record. Harding and Gupta [5] described the defining characteristic of microsimulation models as an analysis of the likely behaviour of and the impact of policy change upon persons (or families, or other microunits). Microsimulation models are often constructed on top of microdata, with sample surveys or administrative data forming typical base datasets for such models.

Microsimulation models are by definition quantitative, typically complex and large. Although there is a broad range of microsimulation models, they have been traditionally divided into two broad categories—static and dynamic [5]. Static models typically use static ageing techniques to update cross-sectional microdata up to the required point in time. Dynamic models simulate the major life events of individuals within the original microdata or base file.

Apart from making a major contribution to the development of tax and transfer policies, microsimulation models in recent years have become more common in the research and policy areas of health and aged care [6]. The models involve pharmaceutical subsidies [7–9], human resource issues in health [10], medical insurance schemes [11], dental health services [12], uncertainty and patient heterogeneity [13, 14], and aged care [15]. Another fast growing research area is spatial microsimulation that focuses on predicting the local effects of policy change and service needs of small area populations [16–18].

Kunming, as the research city in the paper, is the capital of Yunnan Province, located in the southwest of China, with a total population of 6.08 million at the end of 2005 [19]. Kunming commenced its medical insurance reform for urban employees and retirees in April 2001. There were about 1.08 million insured urban employees and retirees at the end of 2005. Like other urban areas in China, the basic medical insurance funds in Kunming, contributed jointly by employers and employees, include the social pool fund and the personal savings accounts. Basically, the social pool fund is mainly responsible for hospitalization expenses, while the personal savings accounts are responsible for outpatient treatment fees.

As one of 79 pilot cities chosen by the Central Government, Kunming began its urban residents' medical insurance scheme in October 2007. The plan aims to provide basic medical insurance for children, students, and adult residents who are out of the labour force. The premiums are paid by households or families, and the governments give subsidies of at least 70 per cent of the insurance premiums annually to each participant, with more going to families with low-income earners and disabled individuals. A medical insurance pool fund has been set up for assisting hospitalization and clinic serious illness treatments. The goal has been set to cover 0.90 million urban residents under the medical insurance scheme in 2009 and to cover all 1.2 million of urban residents in 2010.

Two main data sources were used in the model. The first data are the information on Kunming's population, involving an 0.095 per cent sample of the National Population Census which was conducted in 2000 and results of the 1 per cent Population Survey conducted in 2005. These two kinds of datasets provide the demographic information on urban residents and updated population structures and help to construct the population base file of all urban residents in Kunming.

The sample from the 2000 Population Census conducted in late 2000 was created by the statistical method of selecting households randomly, with a sample rate of 0.095 per cent. The sample contains 5395 individual records of Kunming residents, representing a population of 5.78 million in 2000. At the end of 2005, China conducted a 1 per cent nationwide population survey. According to the report of the survey [20], Kunming had a population of 6.0857 million in 2005. The assembly of this population survey provides benchmarks for updating the population from 2000 to 2005.

The second one is the individual data of medical care records of the urban employees and retirees in Kunming for the period of 2001–2005 and its predicted datasets in 2006–2010. The datasets originally consist of five kinds of medical insurance records for each insured individual, providing the basic information of medical insurance participants as well as the information on the insurance premium contribution records and incurred medical expenses.

This administrative data helped to create a microsimulation model for urban employees and retirees for the period of 2006–2010 [21]. The model evaluated the sustainability of medical insurance scheme for urban employed individuals in China by investigating the balances of the social pool fund and personal savings accounts and predicting the medical expenses shared by different kinds of payment modes.

The administrative medical care records of employed individuals provide for this project the useful personal information as well as comprehensive medical services information. And, as a whole, the dataset of the insured employed individuals is a part of the total urban population of Kunming. In fact, the total urban population is made up of three groups, that is, nonworking residents, employed people with social medical insurance, and employed people without social medical insurance.

As stated above, an urban population model was created using microsimulation model techniques. As the medical insurance scheme for urban residents in Kunming started at the end of 2007, the population model to be created in this research focuses on the period of 2008–2010. The processes of the model mainly involve the following two modules:

- *Population module*: update the 2000 Kunming population census records of residents to the year 2005; estimate the population structures for the period of 2006–2010; then update the population for each year of 2008–2010, respectively.
- *Matching module*: prepare the population of insured individuals in 2005 under the medical insurance scheme for employees and retirees, and statistically match this population with the updated population for urban residents in 2005.

Next two sections will discuss these two modules separately. A population model will be created with the records of the population in 2000 updated first to the target years of 2005–2010. Then, this updated population is then statistically matched with the dataset of the urban employees and retirees. The population distribution and insurance coverage will then be made clear over the period of 2008–2010.

## 2. Population Module

**Figure 1:**
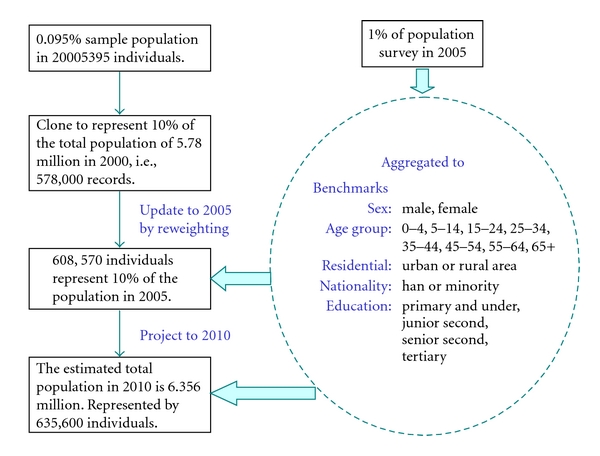
Update the 2000 census data to 2005 and target years.

This section discusses the Population module. In the Population module, the 0.095 per cent sample of the population records in the 2000 Census for Kunming is enlarged (or cloned) to represent about 10 per cent of the total population in 2000. Doing so is to increase the heterogeneity in the final matched dataset, as instead of one person aged 70 having a weight of 10, there will be 10 persons aged 70, each with a weight of 1, and each with a potentially different insurance cover status. Then, using the information compiled from the 1 per cent population survey conducted in 2005, the individual records in the 2000 population sample are reweighted and the population is updated to the year 2005, as Figure 1 shows. The method of the generalised regression estimator reweighting [22] is used to update the population records.

The benchmarks to be used in the population update involve the five variables: sex, age group, residential status, nationality, and education. Then, the population structures are projected to the period of 2006–2010, the target population years, using reweighting techniques.

### 2.1. Sample of the Population Census in 2000

Each person in the population sample in 2000 corresponded to an individual record. Focusing on Kunming's population, the sample contains 5395 individuals' records from 1761 households. The actual population of Kunming reported by the census in 2000 was 5.78 million. In the model, the 2000 population sample is first updated to the year 2005. Then, the updated population dataset is statistically matched with the individual records of employees and retirees who are covered by the social medical insurance scheme.

The total individual records for urban employees and retirees under the social medical insurance scheme are available, as discussed above. In order to keep as much information as possible on individual records, it is planned to match approximately 10 per cent of the population sample with 10 per cent of the insured employees' records. So the 0.095 per cent population sample in 2000 is enlarged to create around a 10 per cent sample of the total population. To do this, the 5395 original individual records were cloned 115 times, and the households' structures were kept unchanged. The new sample has 5395 × 116 = 625,820 individual records. This figure is 10.82 per cent of 5,781,300, the reported population in the 2000 census. Hence, each record in the cloned sample was provided with a weight of 9.23796.

### 2.2. Updating the Population to the End of 2005

At the end of 2005, China conducted a nationwide survey of 1 per cent of its population. According to the survey report, Kunming had a population of 6.0857 million at the end of 2005 [20]. The information gathered from this population survey provided very useful data sources for updating the sample individual records from 2000 to 2005.

The aggregated results of the population in 2005 act as benchmarks in updating the 2000 population census data to the 2005 population records. The generalised regression estimator reweighting techniques [22] are used to update the census individual records. When updating the population records in 2000 to the year 2005, the structures of the households remain the same as in 2000. The benchmark variables in updating the population include sex, age group, residential status, ethnic nationality, and education degrees, which are summarised in Table 1.

In Table 1, the information for the year 2000 is the summed results from the individual sample records of the 2000 population census, while the information in 2005 is summarised from the 2005 population survey [20]. For comparison purposes, the estimated population distributions for the year 2010 are also collated in Table 1. The estimation processes for 2010 will be discussed below.

In processing the population structures in 2005, missing items such as the number of individuals with junior secondary education and number of children aged less than 6 years need to be calculated. As there was no information indicating the proportion with junior secondary education in the 2005 population survey, it assumed it had the same proportion as in 2000 (i.e., 29.97 per cent of the total population aged 6 years or over). The number of children aged less than 6 years was estimated based on the total population and the education distribution, because the survey on education was restricted to people aged 6 years or over.

Regarding the distribution of age groups in 2005, originally only three age groups (i.e., 0–14 years, 15–64 years, 65 years, and over) were available. The detailed information on age groups was estimated using the information in the 2000 Census sample and following the principle of an ageing population. After the age group distribution adjustments, the population of each age group determined (Table 1). 

Based on the population benchmarks in 2005, the individual records in 2000 were updated to the end of 2005. In updating the individual records by using the generalised regression estimator reweighting techniques, a SAS macro called GREGWT was adopted in the model. In the similar way, after updating the 2000 population records to the year 2005, the population units were updated to each year of 2008–2010, respectively, by using the estimated population benchmarks. The benchmarks for the period of 2008–2010 were estimated by using the information collected from the 2000 census and the 2005 survey; this will be discussed in the next section. 

### 2.3. Estimate the Total Population for 2006–2010

As part of the Population module, the projection of the population starts with the total population estimations for each year of 2006–2010, then the population structure for the year 2010, followed by the population distribution estimations for the other four years. 

The target total population for each year of 2006–2010 was estimated first by using the information from the 2000 census and the 2005 population survey. According to the population survey in 2005 [20], the annual natural growth rate (growth due to excess of births over deaths) of Kunming was 0.743 per cent over the period of 2000–2005 (Table 1). However, when using this growth rate, the total population in 2005 was estimated to be under 6 million, which varies from the target population of 6.0857 million. This underestimation possibly occurred because the natural growth rate did not consider the migration population of 86,400. By adjusting the natural growth rate to the overall growth rate (net growth arising from natural increase and net in-migration) of 1.031 per cent and recalculating, the estimated population of 6.0855 million in 2005 was obtained, which is quite close to the target population of 6.0857 million. So the figure 1.031 per cent was assumed to be the population growth rate over the period of 2000–2005. 

Based on the population of 6.0857 million in 2005, the total population in 2006–2010 was estimated. The 2005 population survey concluded “compared with the year 2000, the natural growth rate in 2005 decreased by 0.114 per cent”. So it is assumed in the model that the natural growth rate in 2010 would decrease by a similar proportion compared with 2005, that is, decrease by (0.114% × 1.031%/0.743%) = 0.158%, where 0.743% is the reported natural population growth rate in the 2005 Survey and 1.031% is the adjusted overall growth rate. So the overall population growth rate in 2010 is estimated to be 1.031% − 0.158% = 0.873%.

This growth rate is made to be the average growth rate over 2006–2010. The total population updating is based mainly upon the estimated growth rate. Then, the total population for each year of 2006–2010 can be estimated. It is estimated that Kunming would have a population of 6.3560 million in 2010. 

### 2.4. Estimate the Structure of Population in 2010

After resolving the population estimation for the period of 2006–2010, the population structures on sex, age group, education degree, ethnic nationality, and residential status are determined (Table 1). 

First, the proportion of sex distribution is supposed to follow the results in 2005, with 51.19 per cent of males and 48.81 per cent of females. 

Next, the structure of the age groups is assumed to follow the trend over the period of 2000–2005. The proportion of age group 0–14 years is supposed to reduce 1.27 per cent by the end of 2010 compared with 2005, and the age group 15–64 years in 2010 keeps the same proportion as in the total population as in 2005. Hence, the proportion of people aged 65 years and over is estimated to be up from 8.65 per cent in 2005 to 9.83 per cent in 2010. The estimation of the detailed age group distribution for the year 2010, together with the known population structures in 2000 and 2005, can be used to project the age group distributions for the other years between 2000 and 2010. 

For education, the proportion of children aged less than 6 years was estimated first. Then, following the trends over the period of 2000–2005, the proportions of people with different education categories can be estimated. The 2005 population survey stated that “the population with junior high school education or over has kept increasing and the education for the whole population has further improved”. In the meantime, the proportion of people with primary education was assumed to reduce slightly. Following these principles, the distribution of education degree for individuals aged six years or over in 2010 was estimated. 

Ethnic nationalities are categorised into two groups—the Han nationality and the Minority nationality. Following the trends over the period of 2000–2005, the population of the Han nationality in 2010 is assumed to increase 4.41 per cent compared with 2005, while the Minority population in 2010 is estimated to increase 9.63 per cent compared with 2005. 

For individual's residential status, following the trends of 2000–2005, the proportion of population living in urban areas is assumed to increase from 58.05 per cent in 2005 to 61.14 per cent in 2010. Then, the proportion of population living in rural areas is estimated to be 38.86 per cent in 2010. All the above-mentioned assumptions are summarized in Table 2. 

## 3. Matching Module

**Figure 2:**
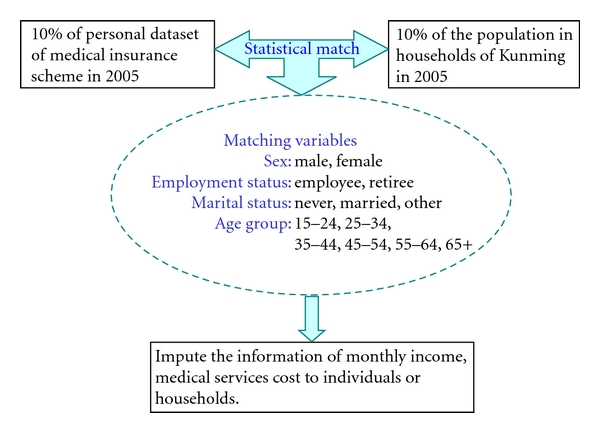
Match the medical insured with the population sample.

 After finishing the Population module, the model then goes to the Matching module. In the Matching module, the individual record dataset for the medical insurance system for urban employees and retirees is statistically matched with the updated total population dataset in households. The first dataset is a sample of the actual individual records of the insured employees and retirees. The second dataset is the updated population records from the above Population module. Both of the datasets are from 2005 and represent around 10 per cent of the corresponding population. The matching variables are sex, age group, employment status, and marital status (Figure 2). 

 As described above, the information gathered from the 1 per cent population survey in 2005 for Kunming acted as benchmarks to update the individual records of the 0.095 per cent sample from the 2000 census to the end of 2005. The updated population records kept the same households' or families' structures as the census sample. This became the base data file that was statistically matched with the individual records of urban employees under the social medical insurance scheme for the same year, which is 2005. 

Statistical matching is a procedure used to link two files or datasets where each record from one of the files is matched with a record from the second file that generally does not represent the same unit but does represent a similar unit [7, 15, 23]. Here, the aim of statistical matching is to add the information of employed status, income, and medical services to the census individual records of urban residents. 

**Figure 3:**
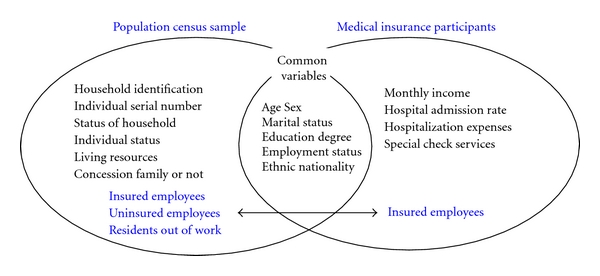
Variables on population census sample and medical insurance participants file.

Figure 3 presents the main variables in the datasets of the population census sample and the Medical insurance participants. The population census sample provides information on household structure, status of household, individual status, and living resources for urban residents. The medical insurance participants' dataset provides information on monthly income and different kinds of medical services. Among the common variables in both datasets, sex, age group, employment status, and marital status act as matching variables when linking the two datasets. As a common variable for statistical matching, employment status is also to be included in the aims of information to be added by statistical matching, by providing the information of income, medical services. 

 The dataset of the population census sample consists of three kinds of populations—insured employees (include retirees), uninsured employees (include retirees), and nonworking residents—while the dataset of the Medical insurance participants only contains insured employees (include retirees). The population of employees (both insured and uninsured) in the census sample is approximately double the population of the medical insurance participants (insured employees). This is because a large number of employees and retirees are not insured under the medical insurance scheme. Therefore, in matching the two datasets, the dataset of medical insurance participants only matches part of the population census sample. 

### 3.1. Basic Population Dataset under Medical Insurance Scheme in 2005

In 2005, the basic dataset under the medical insurance scheme for urban employees in Kunming contained 794,100 individual records. The dataset includes individual information on demography, income, personal savings accounts, and medical treatment expenses. The 2005 basic medical insurance participants' dataset is the primary data file for simulating the medical services of medical insurance participants. 

Apart from the above-mentioned insured employees and retirees numbering 794,100 who were under the administrative of the Kunming Municipal Government, there were two other parts of the population who also came under the social medical insurance coverage. The first one is a group of medical insurance participants in Kunming that is administered directly by the Yunnan Provincial Government (Kunming is the capital city of Yunnan Province). In 2005, this component of the group numbered 282,900. They enjoy the same social medical insurance scheme. However, individual records were not available for this group. Therefore, imputation was used to compensate for the lack of individual records for provincial participants. In detail, 282,900 randomly selected records from the municipal individual dataset in 2005 were used as the individual records managed by the Provincial Government. 

The second group of insured population are senior retirees who are predominantly war veterans and their dependents. This is a special group of people who made contributions to the country before the establishment of the People's Republic of China in 1949. In 2004, their average age was 77 years (ranging from 68 to 101 years). This group of people can have all their medical expenses reimbursed from the local governments [24]. The estimated number of senior retirees in 2005 was 4403. 

It is estimated that the reduction of the population due to death would be higher because their average age of 77 years was greater than the life expectancy of the local residents, which was 74.9 years in 2006 [25]. Because there are no individual records for this population group, their records are created by using the available records under the social medical insurance scheme. Based on restricting the individual dataset of the municipal medical insurance participants in 2005 to individuals aged above 68 years old, 4403 individual records were selected randomly and used to represent the population of senior retirees. 

Combining the above-mentioned three datasets of different types of populations—insured individuals under the Municipal Government, insured individuals under the Provincial Government, and senior retirees—the final dataset in 2005 contained a total of 1,081,403 (= 794100 + 282900 + 4403) insured individuals. 

### 3.2. Statistical Matching of Medical Insurance File and Census File

The total individual records for insured urban employees and retirees are available. In order to keep as much information as possible on individual records, it is planned to match approximately 10 per cent of the population sample with 10 per cent of the insured employees' records. Both of them are for the year of 2005. The 10 per cent of the population sample was enlarged and updated from the 0.095 per cent population sample in 2000 as described in Section 2. 

For the social medical insurance dataset created above, approximately a 10 per cent sample was obtained by using simple random selection, depending on the weights of individuals representing employees and retirees in the census sample file. This sample of the medical insurance dataset contains 97,484 individual records, which represents individuals who are under the social medical insurance scheme for urban employees and retirees. This medical insurance dataset is statistically matched with the updated urban resident population in 2005, compensating for information deficits on household's structure and individual status in either original dataset. Five steps are processed to match the two data files. 

#### 3.2.1. Records Sampling from the Medical Insurance File

Sampling from the medical insurance file in 2005 is used to match the updated population file in 2005. By calculation, it is found that the average weights in the updated population file are: wt2005 = 10.485083034 for employees; wt2005 = 12.542439139 for retirees. 

So for the group of senior retirees, the sampling number should be 4403/12.542439139 = 351 individuals, where 4403 is the number of senior retirees in 2005. The sampling was done for this group by using uniform random numbers to pick up 351 senior retirees. Insured individuals under both Municipal and Provincial administration were put together maintaining the differentiation between employees and retirees. The sampling number for employees and retirees was determined according to the above average weights. Then considering employment status across sex and age group, 68,755 employees and 28,378 retirees were sampled. Together with 351 senior retirees, a total of 97,484 insured individuals were selected randomly. 

#### 3.2.2. Classify Records in Matching Files into Cell Groups

The method used for statistically matching is that the individual records are randomly matched within homogeneous cell groups. These homogeneous cell groups are based on records that have common variables in both of two matched files. The common variables used in this model, called matching variables, include the four variables—sex, age group, employment status, and marital status, as shown in Table 3. The age group is classified into 6 groups which are consistent with the classification of the population survey and the starting age of employment. Based on all the possible combinations of the matching variables, there should be 72 unique cell groups. However, in reality, lower age groups such as those aged less than 34 years are unlikely to contain retired individuals. Therefore, the number of unique cell groups could be less than 72. Records in the same cell group are matched based on a measure of closeness between individual records. 

#### 3.2.3. Select the Match Sample from the Population Census File

For insured individuals in each group cell, the same number of records from the population Census file is selected randomly to match them. Theoretically, the number of population census records in each cell should be greater than the number of insured records, because the latter is part of the former. Actually, most of the cells are as expected, but not for all cells. Adjustments to the closest cell groups for those negative matching numbers were made. Then, a dataset was created by randomly picking up the target number of records (required in each cell group in the insured file) from the population census file. This dataset is ready to be matched with the insured file.

#### 3.2.4. Match Records by Minimising the Distance

The initial and simplest approach is to match each medical insurance record to the closest matching census record. The Mahalanobis distance function [26] was used based on an individual's age. Generally, the selected match minimises the following distance function:

(1) $$d_{i,k}=\sqrt{\frac{\sum_{j}^{}a_{x_{j}}{\left(X_{\text{MIP},i,j,k}-X_{\text{CP},i,j,k}\right)}^{2}}{\sigma_{x_{j},\text{cp}}^{2}}.}$$

The subscript *i* relates to person records, *j* to the matching variable, and *k* to the cell group. *X*_MIP,*i*,*j*,*k*_ is the age of a medical insurance participant, and *X*_CP,*i*,*j*,*k*_  is the age of a population census person. *σ*_*x*_*j*_,cp_^2^ is the variance of the matching variable (here is age). *a*_*x*_*j*__  is the user defined relative importance, or weight given to each matching variable. Because only one matching variable of age was used in the distance function, the value of this weight is one. Also, for the same reason, the variance factor *σ*_*x*_*j*_,cp_^2^ has no impact on the matching distance. Actually, the function simplifies to a simply distance function of *d*_*i*,*k*_ = |*X*_MIP,*i*,*k*_ − *X*_CP,*i*,*k*_|. 

As the two datasets with the exact same group cells and the same number of records in each cell group (based on the common variables: sex, age group, employment status, and marital status) were already created, it is easy to match them by sorting the records by the individual's age in each cell group. And then the corresponding records in the two datasets were matched. After the matching, the average difference of age is only 1 year; the largest age gap in the matched dataset is 20 years, which occurred in the oldest age group of 65 years or over. 99.31 per cent of records have an age gap of less than 4 years, which shows that the matching result is reasonable.

#### 3.2.5. Weighting Adjustment

After matching the population file with the insured file, further adjustments were made to reach the target population of the employed and retired. For example, a big difference occurs between senior retirees in the target population and the estimated population when using the population weights. The target population of senior retirees in 2008 should be 3935, rather than the estimated 5750. The other two years, 2009 and 2010, have the same problem. Because of their old age, the number of the senior retirees is declining with years. The weights for the group of senior retirees were changed using the relative adjusted coefficients for each year over the period 2008–2010, that is,

(2) $$\begin{array}{cc} & \text{New}\;\text{weight}\;\text{for}\;\text{senior}\;\text{retirees}\\ & \;=\text{Old}\;\text{weight}\times \text{Adjusted}\;\text{coefficient}. \end{array}$$

### 3.3. Income Imputation

Because income information has not been provided in the population census data file, the individual's monthly income needs to be projected through imputing income of the insured records onto the population records. Fortunately, the census data file offered some information which could be useful in estimating an individual's income. Individuals who have paid jobs, defined in the census as having worked at least one hour in the week prior to the census, accounted for 53.14 per cent of the total urban population. Children aged less than 15 years accounted for 14.32 per cent of the total population. The remaining 32.63 per cent of the population are residents who are not in the workforce, including students, pensioners, disabled persons, individuals doing housework, and persons looking for work. 

For nonworking residents aged 15 years or over, their living resources largely come from family members (54.36 per cent) or pensions (33.37 per cent). For the rest, living resources include the basic living allowances provided by the governments (3.66 per cent), property income (3.23 per cent), and other income sources (5.38 per cent). When setting the incomes for the individual records, the individuals receiving basic living allowances are assigned 210 Yuan each month as their monthly income [25], so do individuals with property income or other income sources, while the income of individuals who are looked after by their family members are set to zero. 

In processing the monthly income, individuals are categorised into three groups, that is, low income earners, general income earners, and other income earners. It is assumed that if there is at least one person in a family receiving the basic living allowances from the governments, then any employee or retiree in such a family is treated as low income earners. Apart from low income earners, other employees and retirees are treated as general income earners. The general income earners formed a large part of individuals with incomes (94.08 per cent of the total records). The third group, individuals with regular income, is composed of persons receiving the basic living allowances from the governments, persons having income from their property, insurance, or other means. This group of individuals plus the low income earners account for 5.92 per cent of the total income earner records. 

#### 3.3.1. Low Income Earners

Two steps are required to impute income for low income earners. The first step is determining those low income earners in the population of the insured employees in 2008. The second step involves randomly selecting the target number of low income earners, whose monthly incomes are imputed onto the low income earners. By the ratios of low income earners to the corresponding total cell group records, the numbers of corresponding low income earners in the insured population in 2008 are calculated and then are randomly selected by sex across age group across employment status. Consequently, insured records are split into two parts—the low income dataset and the general income dataset. Then, from this low income sampling dataset, the same number of records as the low income earners in the Census dataset is sampled randomly to impute the income for the low income earners.

#### 3.3.2. General Income Earners

For the remaining employees and retirees (apart from the low income earners), their monthly income was imputed using the general income dataset mentioned above for the corresponding year. Similar to the low income earners, factors considered when estimating the income involved sex, age group, and employment status.

#### 3.3.3. Other Income Earners

Other income earners are individuals receiving basic living allowances, having income from their properties or insurances. According to the reported basic living standard by the Kunming Municipal Government [27], the monthly incomes for this group of income earners are consistently assigned 210 Yuan per person in 2008. 

Similarly, income imputations were done for the other two years of 2009 and 2010. The estimated average annual disposable income per person in 2008 is 12,538 Yuan. This is very consistent with the reported figure by the Kunming Municipal Government [14], which was 12,083 Yuan in 2007, with an increase rate of 6.1 per cent compared with the previous year. If this rate of increase of 6.1 per cent is maintained, the annual disposable income per person should be 12,818 Yuan in 2008, which is close to the estimated 12,538 Yuan. 

## 4. Results and Discussion 

### 4.1. Population Distribution Trends

**Figure 4:**
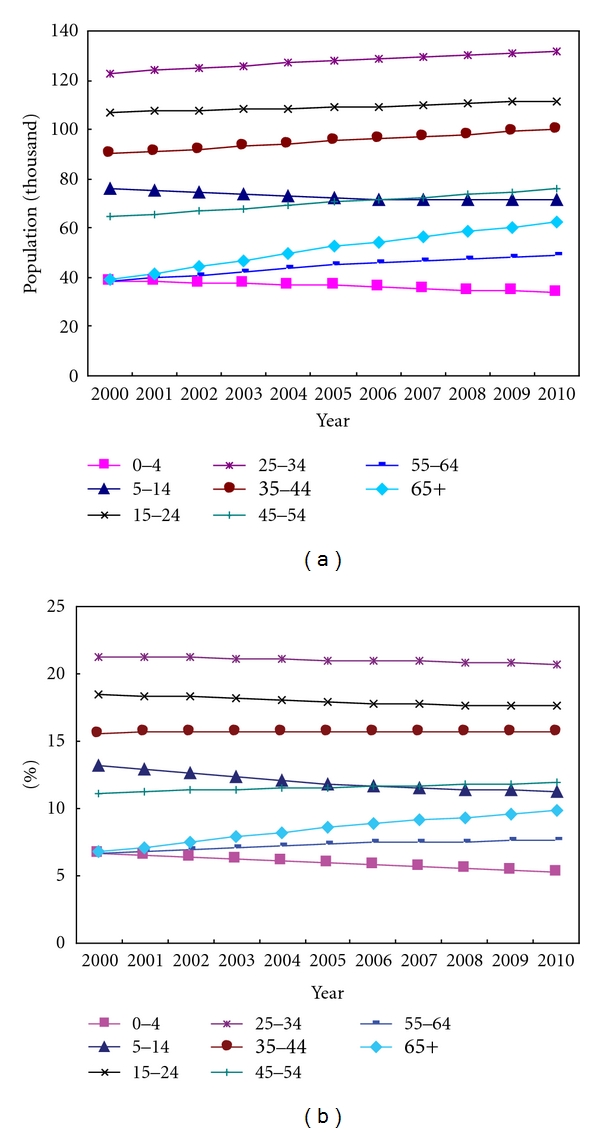
Trends of age groups over the period of 2000–2010. (a) indicates the numbers of the various age groups of population; (b) indicates the percentage of each age group.

Figure 4 presents the trends of the eight age groups over the period of 2000–2010, in which the left side highlights the numbers of the various age group populations and the right side the percentage of each age group. The information for the year 2000 is the summed results from the individual sample records of the 2000 population census, while the information in 2005 is summarised from the 2005 population survey [20]. For the other years, the information was estimated as described in the above sections. From the perspective of both population and percentage, the age groups of 0–4 years (square dot line) and 5–14 years (triangle dot line) show significant downwards trends. The proportion of age groups of 15–24 years and 25–34 years show a slight downward trend, whereas the proportion of people aged over 55 years, especially aged over 65 years (diamond dot line), grows rapidly, indicating that Kunming population is ageing as elsewhere in China. 

Table 4 summarizes the distribution of urban employees and retirees in Kunming city who are covered by the medical insurance scheme for urban employees over the period of 2008–2010. As a comparison, the result in 2005 is also provided in Table 4. It is estimated that a total of 1.27 million individuals would be insured under the medical insurance scheme for urban employees and retirees in 2010. They are administered differently in three ways. The insured individuals under the Municipal administration are about 73.5 per cent of the total insured population. The next largest part of the group consisted of individuals under the Provincial administration, which account for about 26.2 per cent of the total population. The rest and quite small part of less than 0.3 per cent is for senior retirees. The number of insured individuals under the social medical insurance scheme for urban employees is about 33 per cent of the total population in urban areas of Kunming (Table 5). 

Table 5 gives a picture for all the different groups of the population, namely, insured employees, uninsured employees, and residents out of the labour market. Apart from 33 per cent of the insured individuals under the medical insurance scheme for urban employees and retirees, around 36 per cent of the total population are employed or retired but are not covered by the social medical insurance scheme, which is supposed to be a universal plan for all urban employees and retirees. For insured individuals, the ratio of employees to retirees is about 2 : 1, while the relative ratio for uninsured individuals is about 8 : 1. 

### 4.2. Population Distribution of Nonworking Residents

Apart from the insured and uninsured employees and retirees, the remaining population is residents who are out of the labour market and are to be covered by the social medical insurance scheme for urban residents. This group of people is estimated to be 1.18 million in 2008 and 1.22 million in 2010, which accounts for more than 32 per cent of the total urban population. As previously stated, this group of people consists of adults without jobs, students, and children. 

Table 6 focuses on the population of urban residents out of the labour market. The urban residents are categorized into three groups, that is, adults aged 18 years or above, children or students in their primary or secondary education who are less than 18 years of age, and students at the universities. Each category is differentiated into two parts: general and concession. Concession means disabled people or families receiving the basic living allowances from the governments. Children and students less than 18 years of age comprise about 56.6 per cent of the urban residents who are not in jobs. Adult residents account for another 30.5 per cent, and the remaining 12.9 per cent are university students. About a quarter of adult residents are categorised into concession, with children and university students making up 2.82 per cent and 1.47 per cent of the category, respectively. 

**Figure 5:**
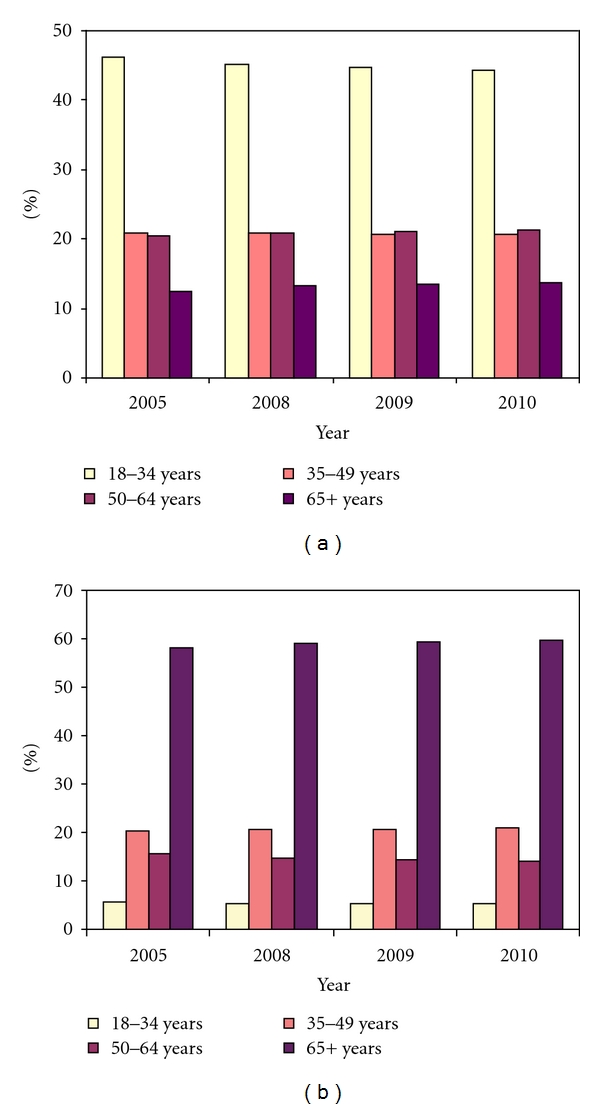
Forecast percentage of age groups of adult residents. (a) for general residents and (b) for concession residents.

Figure 5 presents the proportion estimates for four age groups for adult residents by general and concessional status, with the left side for general residents and the right side for concessional residents. Individuals aged 18–34 years dominate the general residents component, which is about 45 per cent of the total general residents. The other two age groups of 35–49 years and 50–64 years account for 20 per cent each. The remaining 15 per cent of the general population is for individuals aged 65 years or over. 

For concessional residents, elderly people aged 65 years or over account for the greatest proportion of the population, which is close to 60 per cent. This group is followed by individuals aged 35–49 years at 20 per cent, and then 15 per cent are 50–64 years of age, and 5 per cent aged 18–34 years. 

### 4.3. Estimation of Family Income

**Figure 6:**
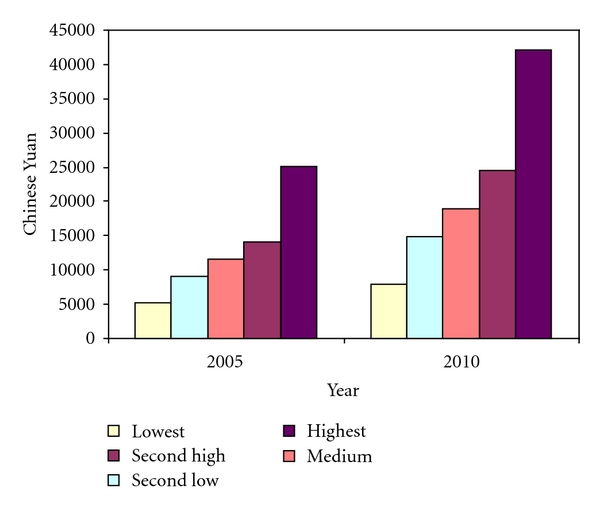
Estimated average annual family income by quintile over 2005–2010.

It is estimated that the middle quintile family income is 15,793 Yuan in 2008 and would increase by about 9.3 per cent in the next couple of years.  Figure 6 illustrates the family annual income by quintile in 2005 and 2010. The family annual income in the highest quintile is 4.71 times the lowest quintile in 2005. The gap between the richest and the poorest is estimated to be wider by 2010, where the relative ratio is 5.34. 

## 5. Conclusion 

 This paper presented on creating a population model for urban employed individuals and nonworking residents using microsimulation techniques. Two main datasets were used in constructing the model—a population sample dataset from the 2000 census for all individuals; a dataset of medical care records of employees and retirees under the social medical insurance scheme. The other information used in the model involves the aggregated results of the 1 per cent population survey of Kunming conducted in 2005. 

Two major steps were conducted to create the model, each of which corresponds to a module. The first step was to project the population structure for the period of 2005–2010 and update the Census sample population in 2000 to 2005–2010, respectively, according to the target population structure. In the second step, the updated census dataset was statistically matched with the individual dataset of insured employees and retirees, which provided information on medical insurance status, monthly income and medical services usage. Consistent with the medical insurance scheme for urban residents which was started in October 2007, the model simulated population structures for the period of 2008–2010. 

In summary, the following achievements have been made in this paper, which help government officials in understanding population structures of Kunming, distribution of different types of population, as well as income trends of units of individuals and families. These factors are essential in making the challenging policy decisions when considering to balance the long-term financial sustainability of the medical insurance scheme. 

First, population structures of Kunming on sex, age group, residential status, nationality, and education were obtained over the period of 2006–2010. The results show that Kunming's population is ageing as elsewhere in China. From the perspective of both population and percentage, the proportion of people aged over 55 years, especially aged over 65 years grows rapidly over 2000–2010, whereas young generation aged 0–14 years shows significant downwards trends. 

Second, the model obtained the distribution of different types of population in the urban area in Kunming, involving insured or uninsured employees, and residents out of the labour force. It was estimated that about 33 per cent of total urban population of Kunming are covered by the medical insurance scheme for urban employees and retirees. This figure only accounts for 47.8 per cent of the population of target urban employed individuals. The remaining 52.2 per cent of employed individuals are not insured under the medical insurance scheme for urban employees and retirees. Such a large percentage of people who are in the labour market but not joining the social medical insurance indicates that a lot of work needs to be done by the local governments to encourage them to be covered by the social medical insurance system. It was found that, for insured individuals, the ratio of employees to retirees is about 2 : 1, while the relative ratio for uninsured individuals is about 8 : 1. These figures indicate that retirees are more eager than employees to join the medical insurance scheme. From another point of view, if the governments can encourage more employees to join the social medical insurance scheme, then it would significantly decrease the risk of running the medical insurance pool fund. Actually, having more elderly persons in the medical insurance pool, thus the scheme would cover a sicker population who are more likely to use medical services insured. But if this can be balanced with increased numbers of younger people within the insurance pool, who are healthier and draw on the resource less as they use less medical services, the insurance scheme is more likely to be sustainable. 

Third, urban residents who are not in the labour force are estimated to be 1.22 million in 2010, which accounts for a little more than 32 per cent of the total urban population. This helps to provide the possible population to be insured in the medical insurance scheme. 

About a quarter of adult residents are categorised into concession, with children and university students making up 2.82 per cent and 1.47 per cent of the category, respectively. Among the concessional residents, elderly people aged 65 years or over account for the greatest proportion of the population, which is close to 60 per cent. This indicates that the elderly people in the concession part, or even adult residents, might be a large part of those using medical services. The elderly people are easier target hit by illnesses or even serious illnesses, so they need more care and financial support from the government. 

Fourth, the incomes of the units of insdividuals and families were estimated. This provides a picture of the average income by individual or family, as well as the gap between the richest and the poorest. For the population records, the individual's monthly income was estimated through imputing income of the insured records for the period of 2008–2010. Individuals who have paid jobs accounted for 53.14 per cent of the total urban population. 

The estimated average annual disposable income per person in 2008 is 14,918 Yuan. The family annual income in the highest quintile is 4.71 times the lowest quintile in 2005. However, the gap between the richest and the poorest is estimated to be wider by 2010, where the relative ratio is 5.34. This indicates that the government needs to pay more attention on low income families when making decisions of medical care scheme. 

Based on this created population model, more work could be done. Different kinds of contributions for the medical insurance premium can be projected. This includes the amount of subsidies by the Central government, the Provincial government, the Municipal government, and universities, as well as the insured individuals. Combined with the other information such as the National Health Services Surveys in 1998 and 2003 [28], it can help to analyse and evaluate the sustainability and the distributional impact of the medical insurance policy settings on urban individuals and families. It is expected to give the resolutions of how the urban poor are likely to be affected by the scheme and how much they have to pay and of whether the contribution rates look appropriate. 

## Figures and Tables

**Table 1:** Population distribution in 2000 and benchmarks in 2005 and 2010.

Items	2000	Percentage	2005	Percentage	2010	Percentage
Total population	5772650		6085700		6355960	
Natural growth rate	0.857%		0.743%		0.629%	
Adjust growth rate			1.031%		0.873%	

Sex						
Male	2994930	51.88	3115270	51.19	3253620	51.19
Female	2777720	48.12	2970430	48.81	3102340	48.81

Age group						
0–4	387340	6.71	365120	6.00	338980	5.33
5–14	762910	13.22	719130	11.82	712930	11.22
15–24	1068940	18.52	1088360	17.88	1117380	17.58
25–34	1230500	21.32	1280240	21.04	1316000	20.71
35–44	900940	15.61	953170	15.66	1001060	15.75
45–54	645210	11.18	704260	11.57	756680	11.91
55–64	384130	6.65	449010	7.38	488140	7.68
65 and over	392690	6.80	526410	8.65	624790	9.83

Residential status						
Urban area	3046230	52.77	3532750	58.05	3886030	61.14
Rural area	2726420	47.23	2552950	41.95	2469930	38.86

Nationality						
Han	5025790	87.06	5265350	86.52	5462370	85.94
Minority	746860	12.94	820350	13.48	893590	14.06

Education						
Tertiary	404200	7.63	455700	8.09	507290	8.55
Senior secondary	778740	14.70	973360	17.28	1178330	19.86
Junior secondary	1587680	29.97	1688170	29.97	1837500	30.97
Primary	2030030	38.32	1994600	35.41	1928280	32.50
Other *	496910	9.38	521040	9.25	481770	8.12
Under 6 years	475080		452830		422790	

Note: *“Other” means illiteracy or semiliterate aged 6 years and over.

**Table 2:** Assumptions of population structures for the year 2010.

Items	Assumptions of proportions
Adjusted growth rate	Estimated to be 0.873%
Sex	51.19% for males, 48.81% for females
Age group	Age group 0–14 years reduce 1.27% compared with 2005, age group 15–64 years keeps the same as in 2005
Education	Junior high school or over keeps increasing, primary education reduces slightly
Ethnic nationality	Compared with 2005, Han nationality increases 4.41%, Minority increases 9.63%
Residential status	Urban living increases from 58.05% to 61.14%, so rural living is 38.86% in 2010

**Table 3:** Matching variables.

Variables	Groups	Explanation
Sex	2	Male, female
Age group	6	15–24, 25–34, 35–44, 45–54, 55–64, 65 and over
Employment status	2	Employee, retiree
Marital status	3	Never married, married, divorced, or other

**Table 4:** Estimated distribution of insured employees and retirees over 2005–2010.

Year	Number of population				Percentage			
	City	Province	Senior	Total	City	Province	Senior	Total
2005	793453	283435	4403	1081290	73.38	26.21	0.41	100.00
2008	885397	315574	3935	1204906	73.48	26.19	0.33	100.00
2009	909780	324153	3774	1237706	73.51	26.19	0.30	100.00
2010	934643	332896	3610	1271149	73.53	26.19	0.28	100.00

Notes: City means under Municipal administration; Province means under Provincial administration; Senior means senior retirees.

**Table 5:** Forecast population distribution of employed and residents.

Year	Insured		Uninsured			Residents*	Total
	Employed	Retired	Employed	Unemployed	Retired		
Population							

2005	720938	360352	1115677	67976	116664	1128020	3441651
2008	803247	401659	1140241	70330	145995	1186056	3677198
2009	825088	412619	1147621	71063	156523	1206081	3747932
2010	847360	423788	1154660	71766	167456	1226169	3819434

Percentage							

2005	20.95	10.47	32.42	1.98	3.39	32.78	100.00
2008	21.84	10.92	31.01	1.91	3.97	32.25	100.00
2009	22.01	11.01	30.62	1.90	4.18	32.18	100.00
2010	22.19	11.10	30.23	1.88	4.38	32.10	100.00

Note: * Residents who are not in the labour force.

**Table 6:** Forecast population and percentage of urban residents out of labour market.

Year	Adults		Children		Uni-students		Total
	General	Concession	General	Concession	General	Concession	
Population							

2005	264146	82409	617370	17949	144026	2121	1128020
2008	274085	88013	651795	18911	150829	2423	1186056
2009	277363	89958	663919	19240	153071	2530	1206081
2010	280606	91931	676264	19569	155160	2639	1226169

Percentage							

2005	23.42	7.31	54.73	1.59	12.77	0.19	100.00
2008	23.11	7.42	54.95	1.59	12.72	0.20	100.00
2009	23.00	7.46	55.05	1.60	12.69	0.21	100.00
2010	22.88	7.50	55.15	1.60	12.65	0.22	100.00
